# Sensory-Driven Development of Protein-Enriched Rye Bread and Cream Cheese for the Nutritional Demands of Older Adults

**DOI:** 10.3390/nu10081006

**Published:** 2018-08-01

**Authors:** Xiao Song, Federico J. A. Perez-Cueto, Wender L. P. Bredie

**Affiliations:** FOOD Design and Consumer Behavior, Department of Food Science, Faculty of Science, University of Copenhagen, Rolighedsvej 26, 1958 Frederiksberg, Denmark; apce@food.ku.dk (F.J.A.P.-C.); wb@food.ku.dk (W.L.P.B.)

**Keywords:** whey protein, soy protein, older consumers, sensory, descriptive analysis, rye bread, cream cheese, protein-enrichment, muscle

## Abstract

To promote healthy aging and minimize age-related loss of muscle mass and strength, adequate protein intake throughout the day is needed. Developing and commercializing protein-enriched foods holds great potential to help fulfill the nutritional demands of older consumers. However, innovation of appealing protein-enriched products is a challenging task since protein-enrichment often leads to reduced food palatability. In this study, rye bread and cream cheese prototypes fortified by whey protein hydrolysate (WPH), whey protein isolate (WPI), and/or soy protein isolate (SPI) were developed. Both sensory properties and consumer liking of prototypes were evaluated. Results showed that different proteins had various effects on the sensory characters of rye bread and cream cheese. The taste and texture modification strategies had positive effects in counteracting negative sensory changes caused by protein-enrichment. Consumers preferred 7% WPH and 4% WPH + 4% SPI-enriched breads with taste and texture modified. Sour taste and dry texture had considerable effects on consumer liking of rye bread. Addition of WPI and butter enhanced the flavor of cream cheese and increased consumer acceptance. Protein-enrichment doubled the protein content in the most liked prototypes, which have the potential to be incorporated into older consumers’ diets and improve their protein intake substantially.

## 1. Introduction

The aging of population is accelerating worldwide, creating a great challenge for societal health care systems [1]. Sufficient intake of a variety of dietary nutrients is required to promote active and healthy aging [2,3,4]. Protein is an essential nutrient to maintain muscle mass and strength during aging. Inadequate protein intake is associated with functional problems such as sarcopenia, which is the age-related loss of skeletal muscle mass, leading to functional decline or even a reduction in independence among 30% of individuals aged above 60 [5,6,7,8]. It has been repeatedly shown that physical exercise and adequate high-quality protein intake throughout the day are two of the most potent stimulators for counteracting the sarcopenic process [5,8,9,10]. Given this background, developing and commercializing appealing protein-enriched foods is a way by which the food industry can assist senior consumers in meeting their nutritional needs [11]. 

High-quality proteins, e.g., soy-based protein and milk-based whey protein, are popular protein supplements to sufficiently support muscle protein synthesis and accretion, since they are all nutritionally complete, highly digestible proteins with high contents and good composition of essential amino acids [12,13]. Consumption of foods enriched with high-quality proteins may not only increase the quantity of protein intake but also potentially improve the quality of protein in the consumer diet through e.g., modifying the amino acid composition of meals. However, the addition of different proteins alters sensory properties of food carriers in different ways, which might lead to reduced palatability for some products or be advantageous for some other products [11,14]. Wendin et al. [11] found that whey protein-fortified muffins were high in bitter taste and astringency mouthfeel and were perceived as very dry in texture. Höglund et al. [14] reported that off flavors were detected for muffins with extra whey protein. Tang and Liu [15] found that the addition of whey protein disrupted the gluten structure of wheat dough and affected cookie texture negatively. On the contrary, soy protein conferred a protective network on partial gluten structure which increased the overall acceptability of the cookies. Soy protein addition to gluten-free bread caused higher crumb hardness, while whey protein-fortified bread had higher crumb porosity [16]. In this context, to develop appealing protein-enriched products, exploring strategies to counteract the disadvantageous sensory influences of protein enrichment is a crucial task. 

Moreover, selecting appropriate food carriers based on the “voice of target consumers” is of great importance for successful innovation of protein-enriched products. It was found that most older consumers perceived the healthy, traditional meal component food carriers as most appropriate for protein-enrichment, which they were most willing to trial purchase as well [17,18]. In the present study, rye bread and cream cheese were chosen for fortification with protein powder, since both rye bread and cream cheese are healthy, traditional foods in Denmark which play an important role in the diet of older Danish adults. Rye bread is one of the most commonly consumed staple foods on a daily basis, especially during breakfast and lunch, primarily among Danish adults. Cream cheese can be combined with meals and a variety of snacks. Through consumption of protein-enriched rye bread and cream cheese, senior consumers could gain a substantial increase in protein intake throughout the day without changing diet habits and meal frequency or size. 

When developing protein-enriched food items for senior citizens, acceptability, which is normally measured in terms of product liking, is of great importance. The correlation between consumer liking and sensory properties could provide developers with a better understanding of product performance and optimization [19]. Consumer acceptance is assumed to be an indicator for prospective purchase intentions and intake as well [19,20]. Furthermore, product-evoked emotions and terms reflecting consumption desire and product satisfaction could be additional measures providing information on how the senior citizen would engage in consuming the product. For instance, product satisfaction could reflect the “confirmation” or “disconfirmation” between consumer expectation and actual food liking, which might influence the final acceptance due to the contrast effect [21]. Food-evoked desire might affect the subsequent food intake [20]. When developing nutrient-enriched products, such parameters may provide additional information beyond liking [19,20,21,22,23,24]. 

This study aims to: (1) compare the effects of whey protein hydrolysate (WPH), whey protein isolate (WPI), and/or soy protein isolate (SPI) enrichment on the sensory attributes of rye bread and cream cheese; and (2) develop protein-enriched rye bread and cream cheese with moderately high protein content and appealing sensory properties. Additionally, older consumers’ liking and product-evoked emotion attributes, including satisfaction and desire, were evaluated to obtain a perspective on consumer experience and engagement in consuming the products. 

## 2. Materials and Methods 

### 2.1. Proteins

The following proteins were applied in this study: soy protein isolate (SPI, 90.0% protein content; Body-kraft, Hørning, Denmark), whey protein isolate (WPI, 87.0% protein content; Arla Foods Ingredients, Aarhus, Denmark), and whey protein hydrolysate (WPH, 86.4% protein content; Arla Foods Ingredients, Aarhus, Denmark).

### 2.2. Rye Bread

#### 2.2.1. Preparation of Rye Bread Sample

In total, 15 rye bread prototypes were developed. Table 1 shows the details of the formulations per loaf of each prototype. The initial dough was prepared mainly according to the guidelines of Amo’s rye bread mix, with additional sunflower seeds added. The amount of initial dough, additional proteins, and/or other ingredients to adjust the bread texture and taste can be seen in Table 1. Numbers in the sample labels indicate the amount of added whey or soy protein (4 = 4%, and 7 = 7%), while T means texture-modified samples and TS represents texture and taste-modified samples.

Wheat gluten, dried sourdough, and water were selected for texture and taste modifications, mainly because they are ingredients that rye bread already contains, thus avoiding too much taste or flavor interference in the bread. Moreover, wheat gluten contains 71.0% protein and sourdough contains 10.0% protein, thus also promoting the protein content of the prototypes. Blends of WPI/WPH and SPI were added in samples WPI 4 + SPI 4 and WPH 4 + SPI 4, respectively. This is because whey protein and soy protein have opposite effects on the texture of rye bread and could potentially counteract with each other’s negative effects on bread texture. During the formula development period, pilot tasting tests were organized in order to investigate the optimal ratio of protein ingredients, wheat gluten, and dried sourdough. Besides, additional sunflower seeds were added to control and protein-enriched breads for improvement of flavor and texture properties, based on results of pilot tests. Moreover, the pH value of leavened bread dough was measured using a handheld pH meter (VWR pH 10, Malmö, Sweden). The heights of baked rye breads were also measured. The data were used to help optimize the addition of dried sourdough, water and wheat gluten for taste and texture modification.

The total weights of all bread dough before baking were the same. The bread dough was prepared and put in 1.2-liter silicone baking tins (length 22 cm/width 8 cm/height 7 cm) to rise at room temperature (around 22 °C) for two hours. Control bread and SPI-enriched breads were baked at 185 °C for 65 min. Breads enriched with WPI, WPH, and blends of whey protein and SPI were baked at 175 °C for 65 min. Breads were weighed before and after baking. The total protein contents per prototype (%) and per slice (g) after baking were calculated and shown in Table 1. Rye breads were cut into 0.85-cm-thick and approximately 2.0 cm × 2.0-cm-sized cubes and put into 60-mL-sized sample cups with lids. Each cup contained two pieces of bread cubes, and each cube had one side of crust. 

#### 2.2.2. Descriptive Analysis of Rye Bread

##### Panelists

The descriptive sensory analysis of rye bread was conducted in the sensory laboratory at the university. In total, 10 screened trained assessors, aged between 23 and 49 years of age, were recruited from the external panel at the Department of Food Science. They had more than one year of experience in sensory evaluation of foods and were familiar with consumption of rye bread. All panelists signed the informed consent of the study and were paid for their participation.

##### Training

Four 2-h training sessions were conducted. In the first session, panelists tasted samples and described odor, appearance, texture, mouthfeel, flavor, and taste of rye breads. They could select attributes from a list of rye bread sensory attributes provided to them, or they could generate new attributes. In the second and third sessions, reference standards of each attribute were presented or defined and discussed by the panelists to select the final sensory vocabulary. In the last session, a final list of odor, appearance, texture, mouthfeel, flavor, taste, and after-taste attributes of the crumb and crust were generated by the panel. Table 2 shows the list of sensory attributes and definitions. Trial assessments of rye breads were conducted in the last two training sessions to confirm that the training was sufficient to ensure clear understanding and proficient judgment of each attribute among panelists. 

##### Assessment

**Figure 1:**
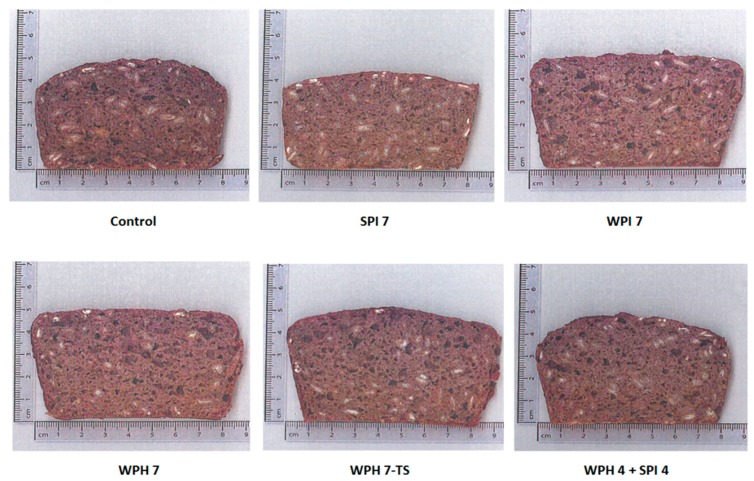
Example photos of cross section of rye bread samples. Labels: numbers indicate the amount of added protein (4 = 4%, and 7 = 7%); TS = texture and taste modified sample; WPH = whey protein hydrolysate; WPI = whey protein isolate; SPI = soy protein isolate.

The 15 rye bread samples were evaluated in quadruplicate in four separate assessment sessions. All assessments were conducted in individual sensory booths at a temperature of 22 °C. Rye bread samples were served at room temperature in a randomized order. Panelists used a 15-cm line scale to rate the perceived intensities of the sensory attributes. Water, cucumber, and plain white bread cubes were provided for mouth cleansing between samples. Two short breaks were held during each assessment session. Photos of the rye bread cross-sections are shown in Figure 1.

### 2.3. Cream Cheese

#### 2.3.1. Recipes of Cream Cheese Prototypes

A total of five cream cheese samples were selected for the descriptive analysis (Table 3). To prepare samples for sensory and consumer evaluation test, ingredients were mixed, put into a 60-mL sample cup and preserved in the refrigerator at 4 °C for more than two hours before being served to assessors. Each sample cup contained 25.0 ± 2.0 g of cream cheese. The total protein content of each prototype (%) and per serving (g) were calculated and are shown in Table 3.

#### 2.3.2. Descriptive Analysis of Cream Cheese

##### Panelists

The descriptive analysis of cream cheese was conducted in the sensory evaluation laboratory at the university. In total, nine trained panelists aged 23 to 29 years old participated in the training and assessment sessions. They were recruited from the screened sensory panel at the Department of Food Science. They had experience of at least one year in the sensory evaluation of foods and were familiar with the consumption of cream cheese. Before the test, all panelists signed an informed consent form for the study. Panelists were paid for their participation.

##### Training

To develop sensory vocabulary of the cream cheese, three 2-h training sessions were conducted. In the first session, panelists tasted samples and generated sensory attributes to describe the cheese. In the second and third sessions, reference standards of each attribute were presented or defined and discussed by the panelists to select the final sensory vocabulary. In the end, the final list of odor, appearance, texture, mouthfeel, flavor, taste, and after-taste attributes of the cream cheese was generated by the panel (Table 4). Trial assessments of cream cheese samples were conducted in the last training session to make sure that panelists had experienced sufficient training to consistently use the attributes to differentiate the products. 

##### Assessment

Cream cheese samples were evaluated in triplicate in three assessment sessions conducted at the sensory evaluation laboratory at a temperature of 22 °C. Cream cheese samples were preserved in a refrigerator at 4 °C for more than two hours before serving to the assessors. All samples were labeled with 3-digit codes and served in randomized order. Panelists used the 15-cm linear scale to rate all attributes of each sample. Water and plain crackers were provided for mouth cleansing between samples. 

### 2.4. Consumer Test

The consumer panel consisted of 72 independent older Danish adults (44 females and 28 males; aged 61 to 83 years old) recruited from the external consumer panel of the Department of Food Science. A consumer acceptance test was conducted in individual sensory booths. The test included two sessions for rye bread tasting and cream cheese tasting, respectively. A 15-min break was held between the two sessions. 

Based on the results of the descriptive analysis, six rye bread prototypes and all five cream cheese prototypes were selected and included for consumer evaluation. Rye bread samples were preserved and served at room temperature. Cream cheese samples were preserved at refrigerator at 4 °C for more than two hours before serving to the test persons. Samples were labeled with three-digit codes and served in a randomized order. Water, cucumber, and plain white bread cubes were provided for mouth cleansing between samples. Each sample was tasted and then evaluated for overall liking and selected product-evoked emotions, which included satisfied, desire, happy, interested, pleasant, calm, disgusted, unhappy, bored, and disappointed. The 9-point hedonic scale [25] was used to measure overall liking (1 = extreme dislike, 5 = neither like nor dislike, 9 = extreme like). The rate-all-that-apply method (RATA) was applied for emotion evaluation. Consumer participants ticked emotions they felt after tasting and rated the intensity of ticked emotions using a 5-point Likert scale (1 = slightly, 3 = moderately, 5 = extremely) [26]. The emotion attributes which were not checked represented emotions that consumers could not feel and were recorded as “0 points”. Consumer demographic characters were also collected, which included age, gender, self-reported health status, living status, education level, and consumption frequency of rye bread and cream cheese. Consumers’ perceived healthiness and willingness to trial purchase protein-enriched rye bread and cream cheese were evaluated using a 5-point Likert scale (1 = not at all, 3 = moderately, 5 = extremely). Before the test, all participants signed the informed consent of the study. After the test, each consumer participant received a goodie bag as the reward.

### 2.5. Data Analysis

The descriptive analysis panel data were analyzed by mixed model analysis of variance (ANOVA) to investigate the significance of each sensory attribute in discriminating products. Products were treated as the fixed factor, and panelists and replications were set as random factors [27]. Principal components analysis (PCA) was performed on average sensory data to relate rye bread and cream cheese products with sensory attributes, respectively. External preference mapping (PREFMAP) was conducted to investigate relationships among consumer acceptance and sensory attributes across rye bread and cream cheese products, respectively. Both PCA and PREFMAP were applied to the significant sensory attributes. Agglomerative hierarchical clustering analysis (AHC) was carried out to investigate the existence of homogeneous clusters of consumers with similar acceptance of rye bread or cream cheese, respectively. One-way ANOVA was conducted on consumer liking data and emotion data with post hoc Fisher’s least significant difference (LSD) test. A penalty-lift analysis [28,29,30,31] was performed to analyze emotion RATA data in relation to the liking scores. The XLSTAT version 2018.3 (Addinsoft, New York, NY, USA) and SPSS statistics version 24 (IBM, Armonk, NY, USA) software packages were used for data analysis. 

## 3. Results

### 3.1. Sensory Descriptive Analysis of Rye Bread 

Table 2 presents the list of the 29 sensory attributes of rye bread assessed by 10 trained panelists in four replicate sessions. The attributes covered the odor, appearance, mouthfeel, texture, flavor, taste, and the after-taste of crumbs and crust. From an ANOVA analysis of the sensory data, it was found that all attributes, apart from the crumb’s beany flavor, grainy flavor, salty taste, and bitter taste, were significantly different (*p* < 0.05) across the rye bread samples tested. This indicated that most of the sensory attributes were useful in characterizing differences across bread samples. Attributes which were not significantly different among rye bread prototypes were not included in further PCA analysis.

**Figure 2:**
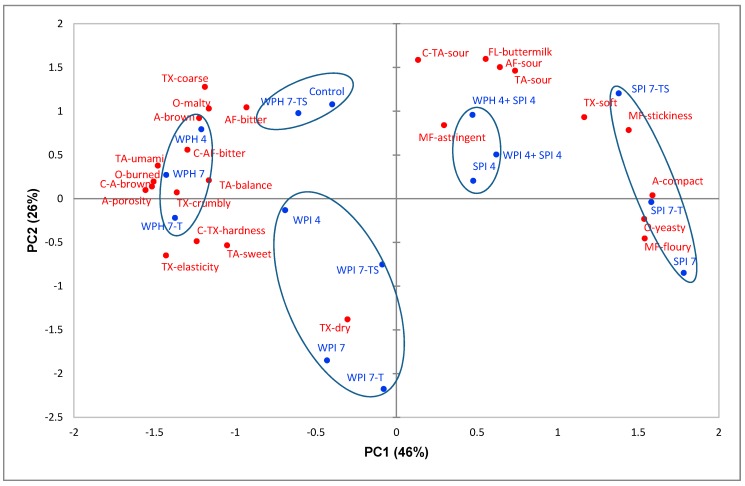
Principal component analysis bi-plot of sensory attributes (red labels) and rye bread samples (blue labels). PC1 = first principal component; PC2 = second principal component. Red labels: sensory attributes; C = attributes for crust; attributes without “C” are attributes for crumbs; O = odor attributes; A = appearance attributes; TA = taste attributes; TX = texture attributes; FL = flavor attributes; MF = mouthfeel attributes; and AF = after taste attributes. Blue labels: rye bread samples; numbers indicate amount of added protein (4 = 4%, and 7 = 7%). T = texture-modified samples; TS = texture and taste-modified samples.

The relationship between rye bread samples and significant sensory attributes were visualized by principal component analysis (PCA). Figure 2 presents the PCA bi-plot of sensory attributes for all 15 rye bread samples. The first two principal components (PCs) accounted for 72% of the total variance (46% for PC1, 26% for PC2). PC1 separated the bread samples mainly according to yeasty odor, the crumb’s compact appearance, and floury and sticky mouthfeel in the positive direction, and burned odor, the crumb’s porous appearance, crumbly texture, and umami taste, and the crust’s brown appearance, hard texture, and bitter after-taste in the negative direction. PC2 was positively linked with the sour taste, sour after-taste, and buttermilk flavor located in the positive direction and was negatively associated with the dry texture.

The PCA bi-plot shows that the sample groups spanned the sensory space quite well (Figure 2). WPI-enriched samples (WPI 4, WPI 7, WPI 7-T and WPI 7-TS) were closely linked to dry texture, and negatively related with astringent mouthfeel, sour taste, sour after-taste, and buttermilk flavor. WPH-enriched breads (WPH 4, WPH 7, WPH 7-T, and WPH 7-TS) were characterized by a brown and porous appearance, burned odor, crumbly and elastic texture, umami taste, and bitter after-taste. Moreover, they had a negative correlation with the attributes of yeasty odor, compact appearance, and floury mouthfeel. The three 7% SPI-enriched samples (SPI 7, SPI 7-T and SPI 7-TS) correlated with a yeasty odor, compact appearance, soft texture, and floury and sticky mouthfeel. The 4% SPI-enriched bread (SPI 4) and breads enriched by mixed proteins (WPH 4 + SPI 4 and WPI 4 + SPI 4) were characterized by sour taste, sour after-taste, buttermilk flavor, astringent mouthfeel, and soft texture. Compared with WPI and SPI, WPH-enriched samples were located much closer to the control sample, which demonstrated that WPH enrichment altered the sensory properties of rye bread to a smaller extent. Thus, WPH could be regarded as a more appropriate protein type for enrichment in rye bread in this study.

To compare the effects of different protein types on bread sensory characters, it was found that most WPH-enriched samples were located close to burned odor and brown appearance, which could be explained by the enhanced Maillard reactions because of the addition of WPH [32]. Moreover, the three 7% WPI-enriched breads had a less soft and drier texture. The textures of WPH 7 and WPH 7-T were more crumbly, hard, and elastic. Furthermore, compared to the remaining samples, the four WPH -enriched rye breads had higher umami taste and bitter after-taste, which is in line with prior research [32,33,34]. Crumbs of the three 7% SPI-enriched breads appeared more compact and less porous, and had more floury and sticky mouthfeel and less crumbly texture- (Figure 2). 

In terms of influences from taste and texture modification strategies, it was found that compared with WPH 7 and WPH 7-T, sample WPH 7-TS was located much closer to the control sample. This indicated that the taste and texture modification strategies (addition of gluten and sourdough) reached positive effects in counteracting adverse sensory changes caused by the WPH-enrichment. The enrichment of higher percentage of all three kinds of proteins decreased the buttermilk flavor and sour taste, which could be explained by the increased pH value due to protein enrichment. The control sample had pH value of 4.0, while the average pH values of SPI 7, WPI 7, and WPH 7 were 4.6, 4.8, and 4.8 (data not shown), respectively. The addition of dried sourdough adjusted the sour taste in samples SPI 7-TS, WPI 7-TS, and WPH 7-TS so that they had a sour taste intensity closer to that of the control sample. 

Moreover, it could be observed that whey protein and soy protein had opposing influences on the texture characters of rye bread. It was found that the addition of 4% SPI to 4% WPI or 4% WPH-enriched rye bread reduced the crumbly texture and increased the soft texture successfully. Enrichment with the blend of WPI/WPH and SPI not only counteracted each other’s effects on bread texture but also resulted in an increase in the total amount of additional protein. 

In summary, texture and taste modification strategies had positive effects in counteracting negative sensory changes caused by the protein-enrichment, especially in correcting the crumbly texture, compact appearance, floury mouthfeel, and/or sour taste of breads enriched with the higher percentage of proteins. WPH was found to be the most appropriate ingredient for rye bread enrichment. The 7% WPH-enriched, texture and taste-modified rye bread sample (WPH 7-TS) was the optimal sample, showing little sensory difference with respect to the non-enriched control bread. 

### 3.2. Consumer Liking of Rye Bread

**Figure 3:**
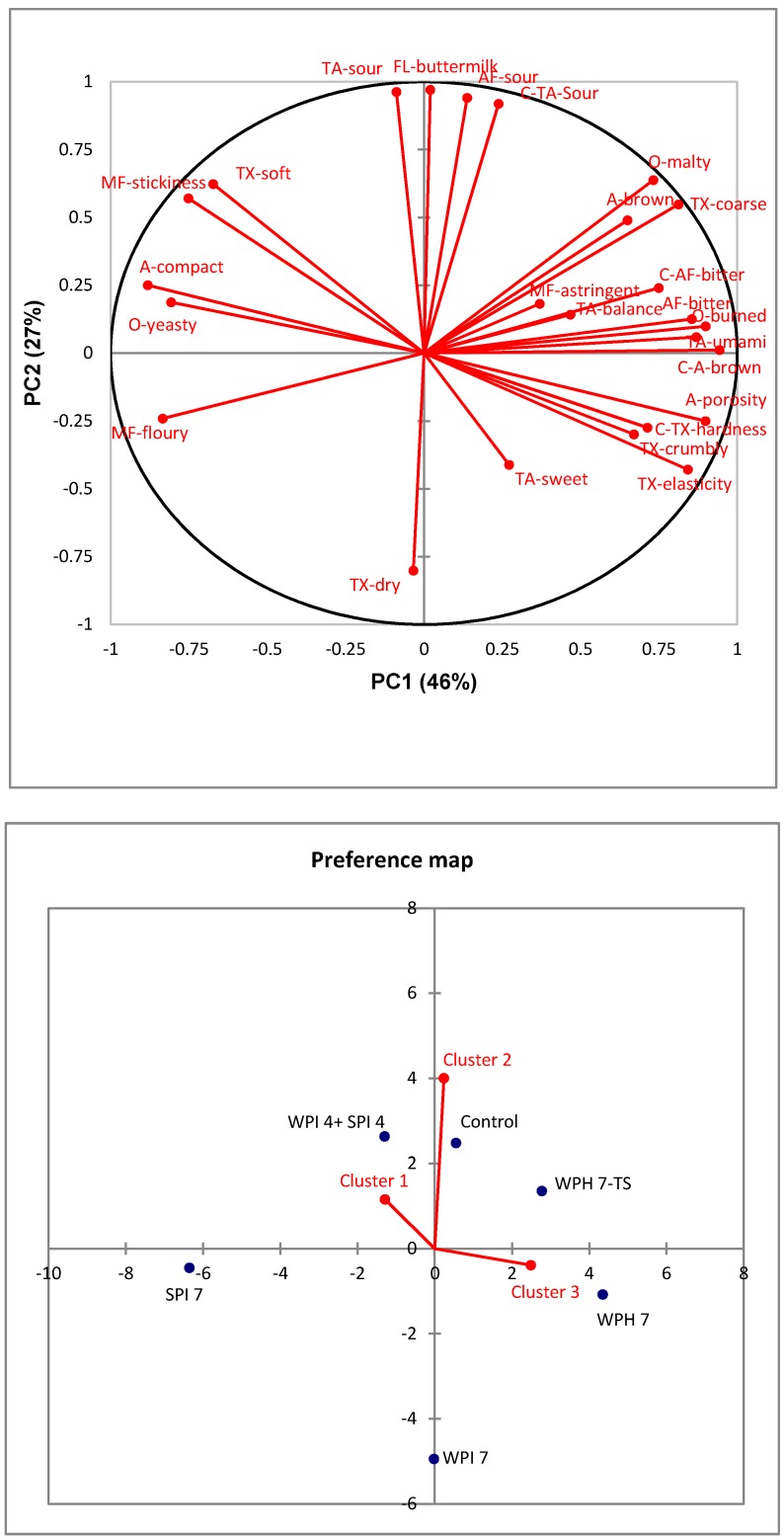
External preference mapping of rye bread. Red labels: O = odor attributes; A = appearance attributes; TA = taste attributes; TX = texture attributes; FL = flavor attributes; MF = mouthfeel attributes; AF = after-taste attributes. Blue labels: numbers indicate amount of added protein (4 = 4%, and 7 = 7%); TS = texture and taste-modified samples.

In total, six bread samples (control, WPH 7-TS, WPH 7, WPI 7, SPI 7, WPH 4 + SPI 4) were chosen for consumer evaluation based on the results of sensory descriptive analysis. The sensory space spanned by the 15 rye bread samples (Figure 2) was well-represented by the six bread samples selected for the consumer acceptance test. The average ratings of consumer overall liking of rye breads are shown in Table 5. Consumers who were homogenous in their acceptance towards different rye bread samples were grouped through agglomerative hierarchical clustering (AHC). Figure 3 represents the external preference mapping to demonstrate the correlation between sensory attributes and the overall liking of different consumer clusters, with average sensory data as the explanatory variables (X) and mean liking ratings of three consumer clusters as responses (Y). The mean liking ratings of consumer clusters are also shown in Table 5. 

Table 5 showed that the average overall liking ratings of each bread sample ranged from 5.5 to 6.5. Significant differences (*p* < 0.05) of consumer overall liking were found across the sample of six rye breads. WPH 7-TS rye bread (6.0) and WPH 4 + SPI 4 (5.9) were the most accepted protein-enriched samples, amongst which WPH 7-TS showed no significant difference in terms of consumer acceptance compared to the control bread (*p* > 0.05). Moreover, the taste and texture modification of WPH 7-TS increased consumer liking by 0.4 units compared to WPH 7 (5.6). SPI 7 and WPI 7 were the least preferred rye bread samples, with significantly lower liking ratings compared to the other four samples (*p* < 0.05). 

The external preference mapping plots are presented in Figure 3. AHC identified three consumer clusters representing different patterns of product liking. In cluster 1, 24% of the consumers were located relatively close to the WPH 4 + SPI 4 and control samples, which had significantly higher overall liking ratings (6.3 and 6.7, respectively) as compared to the remaining four samples in cluster 1. The attributes sour taste, soft texture, and sticky mouthfeel which characterized the control and WPH 4 + SPI 4 appeared to influence the consumer liking of cluster 1 positively. WPH 7 and WPH 7-TS, with hard, crumbly, and elastic textures were the least liked in cluster 1. Cluster 2 (50%) liked the control sample (6.8) the most, which was characterized by sour taste, sour after-taste, and a sour-related buttermilk flavor. Cluster 2 showed the least preference towards sample WPI 7 (4.7), which had a dry texture. Consumers of clusters 1 and 2 (74%) could be regarded as ‘sour rye bread lovers’. Consumers in cluster 3 (26%) liked sample WPH 7-TS (6.6) the most and sample SPI 7 (4.4) the least. It appeared that they were attracted by WPH 7-TS with its brown (crust) and porous appearance, burned odor, crumbly and hard (crust) texture, and umami and bitter after-taste, and disliked SPI 7, with its yeasty odor and compact appearance. Thus, it seems that the sourness levels and texture and mouthfeel properties of rye bread might play important roles in influencing the liking of most consumers. Demographic characters were compared across three clusters as well and no significant differences were found. The mean rating of consumers’ willingness to trial purchase protein-enriched rye bread was very high (4.0 on the 5-point Likert scale).

### 3.3. Sensory Descriptive Analysis of Cream Cheese

Table 4 shows the list of the descriptive sensory attributes of cream cheese assessed by the nine trained panelists in triplicate. The attributes characterized the odor, appearance, mouthfeel, texture, flavor, taste, and after-taste aspects of cream cheese. The attributes meltdown rate, coating mouthfeel, astringent mouthfeel, egg yolk flavor, and salty, sweet, and umami tastes were not significant in discriminating cheese products (*p* > 0.05), and thus were excluded in further PCA and PREFMAP analyses. Cheese prototypes enriched with SPI were not included in the sensory descriptive analysis due to their poor sensory performance compared with WPI- and WPH-enriched samples. 

**Figure 4:**
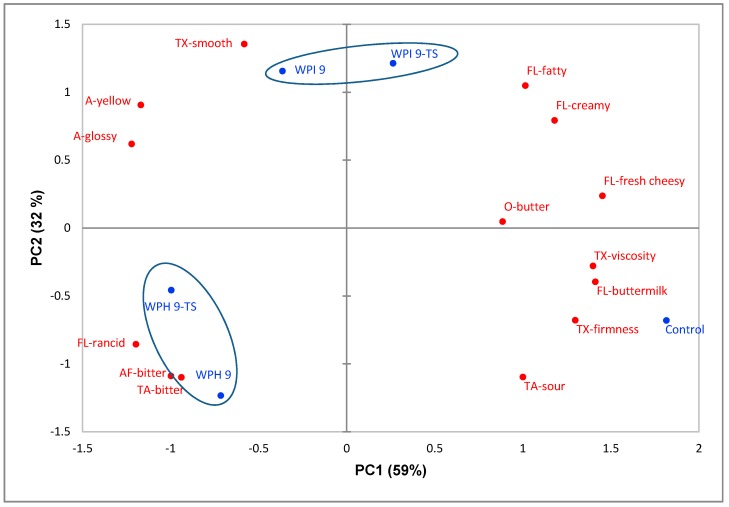
Principal components analysis bi-plot of sensory attributes (red labels) and cream cheese samples (blue labels). Blue labels: numbers indicate amount of added protein (9 = 9%); TS = texture and taste-modified samples. Red labels: O = odor attributes; A = appearance attributes; TA = taste attributes; TX = texture attributes; FL = flavor attributes; AF = aftertaste attributes.

Figure 4 shows the PCA bi-plot of sensory attributes for five cream cheese samples. The first principal component accounted for 59% of the total variance, while the second principal component explained 32% of the total variance. The first two PCs explained 91% of the total variance. The non-enriched control sample is loaded in the fourth quadrant. It was characterized by buttermilk flavor, sour taste, and firm and viscous texture, and was negatively linked with a yellow and glossy appearance. The 9% WPI-enriched, butter-added sample (WPI 9-TS) is loaded in the first quadrant and was closely correlated with fatty, creamy, and fresh cheesy flavors. WPI 9 was associated with smooth texture, yellow appearance, and glossy appearance in the second quadrant. The two samples enriched by 9% WPH (WPH 9 and WPH 9-TS) are located most closely to the less-desired rancid flavor, bitter taste, and bitter after-taste in the third quadrant. All protein-enriched samples, except WPI 9-TS, are loaded in the left side of the map. 

The increased glossiness of cheese samples due to protein enrichment could be explained by the texture changes: increased smoothness and decreased firmness and viscosity. Some panelists used *watery* to describe the surface of protein-enriched cream cheese during the profiling session. The decreased firmness and viscosity might be due to the effects of whey protein on the oil/water emulsion, which led to a decreased extent of partial coalescence and increased extent of fat destabilization [35]. The increased yellowness of protein and/or butter-enriched cheese might be explained by the light-yellow color of dissolved protein powder and/or the higher fat content and larger fat droplets of the cheese [36]. The bitter taste and rancid flavor in WPH-enriched samples could be explained mainly by bitter peptides and some off-flavor compounds in WPH, respectively [37]. Moreover, in contrast to rye bread, the addition of WPH had no significant influence on the umami taste, which could be because the cheesy and creamy flavor masked the umami taste to a large extent. Regarding the taste and texture modification, it appeared that addition of 10% butter in the WPI 9-TS sample helped with the improvement of flavor [36] but not enough to counteract the softening texture effect from protein fortification completely. 

In summary, WPI was more adequate for use in cream cheese enrichment when texture and taste treatment was applied, as compared to WPH. The texture and taste modification strategy achieved positive effects in enhancing pleasant flavors in cream cheese. 

### 3.4. Consumer Liking of Cream Cheese

**Figure 5:**
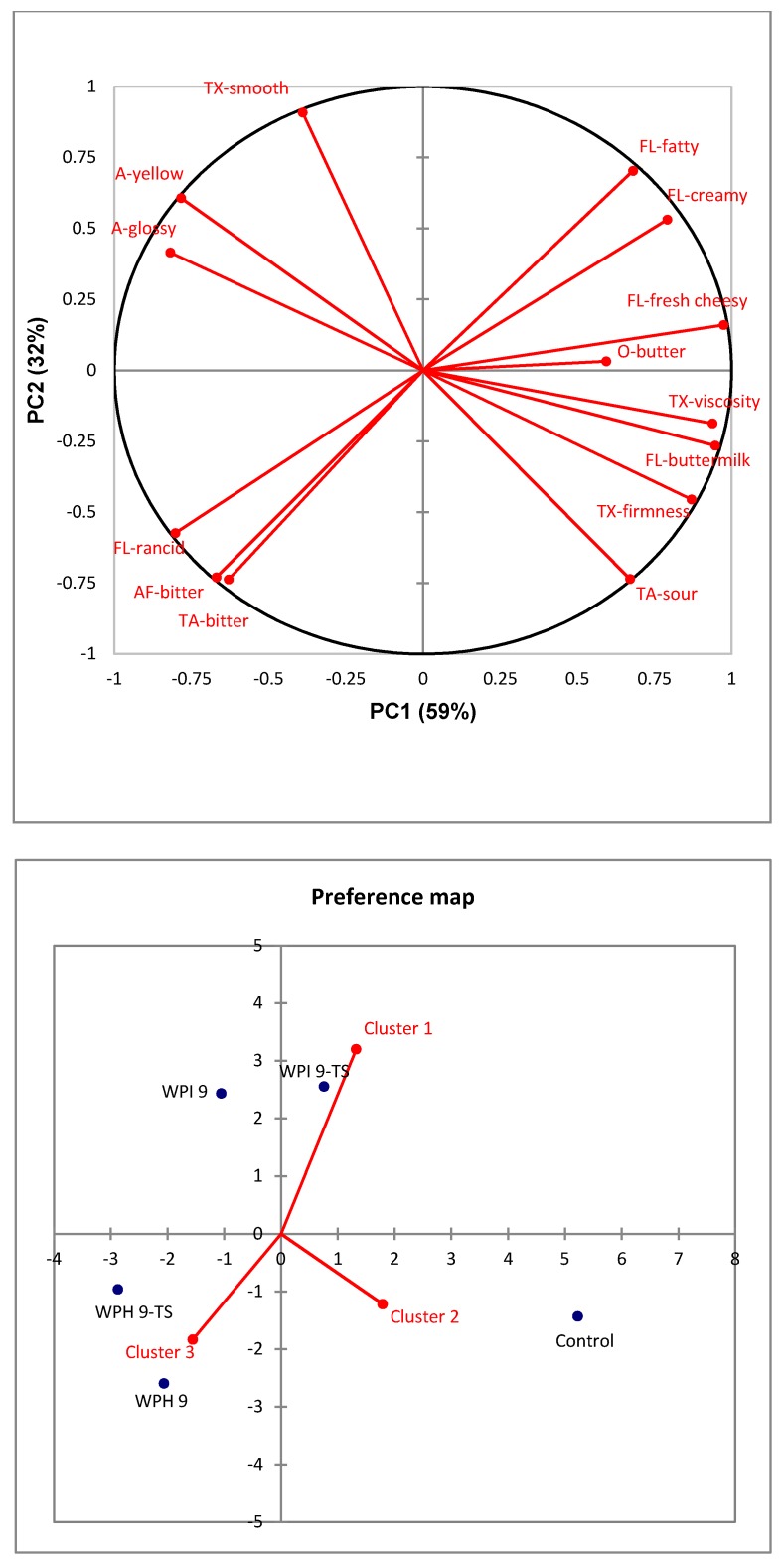
External preference mapping of cream cheese. Red labels: O = odor attributes; A = appearance attributes; TA = taste attributes; TX = texture attributes; FL = flavor attributes; AF = after taste attributes. Blue labels: numbers indicate amount of added protein (9 = 9%), TS = texture and taste-modified samples.

The average ratings of consumer liking are shown in Table 6. For a better understanding of consumer preference, agglomerative hierarchical clustering (AHC) was conducted for a group consumers with a similar acceptance towards different cheese samples. The mean liking ratings per cluster are also shown in Table 6. The external preference mapping plots are presented in Figure 5, which allows a visual representation of the association between cheese samples, sensory attributes, and consumer liking of each cluster. 

In Table 6, significant differences (*p* < 0.05) in overall consumer liking were found among the five cheese samples. Acceptance of WPI 9-TS and the control sample was significantly higher than the two WPH-enriched samples (*p* < 0.05). Sample WPI 9-TS was the most liked protein-enriched sample. Besides, acceptance values of WPI 9-TS (6.9) and control sample (6.3) were not significantly different (*p* > 0.05). Compared to cheese enriched with 9% WPI (6.1), the addition of butter in WPI 9-TS successfully enhanced the flavor and increased consumer liking significantly (*p* < 0.05). 

The external preference mapping plots of cream cheese are presented in Figure 5. Agglomerative hierarchical clustering identified three consumer clusters. Cluster 1 was the largest group, accounting for 68% of total consumers. This cluster was located in the first quadrant and consumers most liked WPI 9-TS (7.2) characterized by a fatty, creamy and fresh cheesy flavor and butter odor. WPH 9 (5.1), with a rancid flavor and bitter taste, was liked the least by consumer cluster 1. Cluster 2 (24%) expressed the highest liking towards the control sample (7.1) characterized by firmness, viscosity, and a buttermilk flavor, and lowest liking ratings were for WPH 9-TS (5.5), with a bitter taste and rancid flavor, and WPI 9 (5.8), with a yellow and glossy appearance. Cluster 3 (8%) was a small cluster characterized by consumers who liked WPH 9-TS, with a bitter taste, bitter after-taste, and rancid flavor. This might be because a small percentage of older adults may not be sensitive to bitter taste [38]. Demographic data were compared across three clusters, but no significant differences were found. Consumers had moderately high willingness (rated 3.6 on the 5-point scale) towards consumption of protein-enriched cream cheese in general.

The bitter taste and rancid flavor of WPH restricted its application in cream cheese. WPI-enriched cream cheese with additional butter was regarded as the most promising prototype for its outstanding performance in both sensory and consumer liking evaluations. 

### 3.5. Product-Evoked Emotions 

**Figure 6:**
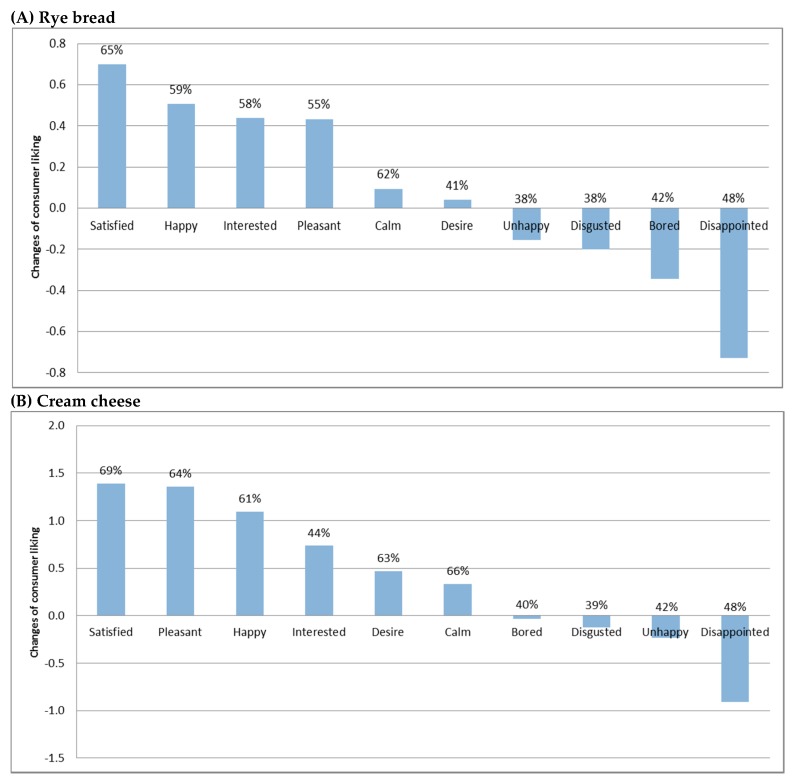
Penalty-lift analysis of emotions’ effect on overall liking across (**A**) rye bread samples and (**B**) cheese samples. The frequency (%) of which the emotion descriptors were checked by consumers is also indicated. The values of the vertical axis indicate the unit of change in liking of prototypes for which the respective emotion attribute was checked, compared to liking of prototypes for which the emotion attribute was not checked. The upstand pillars represent the increase in consumer liking and the downward pillars indicate the decrease in consumer liking.

The penalty lift analysis [28,29,30,31] was performed to demonstrate the extent that product-evoked emotions affected consumer liking acquisition. All emotion words were applied by more than 20% of consumers; thus, all were included in the analysis [28]. Figure 6 shows the results of penalty-lift analysis of rye bread and cream cheese-evoked emotion data. The elicited positive emotions led to increased consumer liking, and negative emotions indicated reductions of consumer liking, which was in line with former research [31]. 

Satisfaction and disappointment represent the gap between consumers’ expected liking and experienced liking. The degree of satisfaction indicates the extent that experienced liking goes beyond expectations, while disappointment means the experienced liking does not meet with consumer expectations. Lower expectation and higher experienced liking result in higher satisfaction and lower disappointment. In this study, the liking ratings of satisfied consumers were 0.7 unit and 1.4 units higher than all consumers’ average liking ratings of rye bread and cheese, respectively. Disappointment was the most detrimental emotion for liking acquisition of both food matrixes, which reduced the liking ratings for rye bread and cream cheese by 0.7 units and 0.9 units, respectively. 

To further investigate the discrimination power of emotions across six rye bread samples and five cream cheese samples, analysis of variance (ANOVA) was also performed on the ratings of emotion descriptors. Results indicated that satisfied, happy, and disappointed performed significantly better in discriminating rye bread samples (*p* < 0.05), whilst satisfied, pleasant, and disappointed went beyond the remaining emotions for discriminating cheese samples (*p* < 0.05). 

Moreover, it should be noted that consumers checked desire for cream cheese much more frequently (63%) than rye bread samples (41%). This might be because high-fat foods usually stimulate higher desire to eat and high-fiber and carbohydrate foods often evoke lower consumption desire [39]. The degree of desire often affects the subsequent food intake [20]. To design protein-enriched meals which could stimulate stronger desire and more subsequent food intake, a combination of high-fat foods with high-fiber and carbohydrate foods could be a good option. 

In summary, for the two food matrixes, the satisfaction-related emotions satisfied and disappointed were among the most influential emotions on both product liking and discrimination among older consumers. Furthermore, besides experienced liking, desire and satisfaction/disappointment could be useful measurements to indicate prospective food intake, which may guide the design of protein-enriched dishes and meals [24].

## 4. Discussion

In this study, we explored the sensory and consumer acceptance changes caused by enriching rye bread and cream cheese with whey protein hydrolysate (WPH), whey protein isolate (WPI), and/or soy protein isolate (SPI). Descriptive analysis results showed that different proteins had various influences on the sensory performance of the two food matrices. Consumers with homogenous acceptance towards rye bread and cream cheese were grouped into their respective clusters. The sensory attributes driving the liking of each consumer cluster were demonstrated. 

WPH enrichment led to higher bitter after-taste in rye bread, mainly due to the increased Maillard reaction and content of bitter peptides [30,34]. However, PREFMAP of rye bread showed that bitterness seems had no negative effect on the acceptance of most consumers; a small group of consumers even appeared to be attracted by the bitterness of rye breads. This might be because bitter taste is a typical sensory character in rye bread [40]; even though WPH increased bitter after-taste to some extent, the intensity was not beyond the accepted level among senior consumers. Moreover, the sour taste and sour-related flavor seem to be important in affecting the acceptance among most consumers, which explained the high liking towards WPH 7-TS. It was also noted that WPH increased the umami taste, which might be elicited from the free amino acids released during the hydrolysis of protein [41,42]. This could have advantageous effects in food matrices requiring an umami taste, e.g., a variety of soups and sausages. Regarding the texture changes caused by WPH- and WPI-enrichment, the increased hardness and elasticity could be explained by the heat-induced aggregation of whey protein [43,44]. The foaming property of whey protein may lead to the porous appearance, larger volume, and crumbly texture [45]. The high water-binding capacity of whey protein might be the reason which increases the perceived dryness during mastication [11].

Isolated protein (WPI and SPI)-enriched rye breads had a lower bitter taste compared to WPH. However, the texture and/or mouthfeel of WPI and SPI-enriched breads restricted their application in rye breads. The dry texture among WPI-enriched breads might be the major problem that led to the breads being disliked by at least half of the consumers. SPI enrichment increased the stickiness, floury mouthfeel, and compactness of rye breads, which appeared to decrease the acceptance of more than half the consumers. The sensory changes caused by SPI might be because the soy protein conferred a protective network on partial gluten structure which increased the dense and sticky texture of the dough and bread [15,46]. Enrichment with the blend of WPI/WPH and SPI counteracted each other’s effects on rye bread texture and contributed to consumer liking. 

In contrast to rye breads, when applying WPH in cream cheese, the increased bitter taste and rancid flavor appeared to reduce consumer acceptance significantly. WPI was regarded as more proper for cream cheese enrichment when additional butter was added for flavor enhancement. The flavor advantage of WPI 9-TS cream cheese might be the major reason explaining its higher liking rating. 

The sour taste seems dominate consumer liking in rye breads. More diversity was found in consumer liking towards bread texture/mouthfeel. At least half consumers disliked WPI 7 with dry texture; a quarter of the consumers liked WPH samples characterized by a crumbly texture. The remaining one-quarter of consumers appeared to be attracted by WPH 4 + SPI 4 with sticky mouthfeel and soft texture to some extent. For cream cheese, the liking of most consumers (68%) seems to be mainly affected by the odor and the flavor dimension, which led to the high liking of WPI 9-TS cheese. The liking of the remaining consumers appeared to be dominated by appearance, texture, and flavor aspects, amongst which the viscosity, firmness, and buttermilk flavor which characterized the control sample attracted the most consumers in this group. Compared to cream cheese, the variety in texture preferences towards rye breads could be due to the texture complexity of the products. Moreover, individual differences in the ability or preferred way to manipulate food in their mouth could also contribute to the diversity in texture preference, as shown in a recent study on texture mouth behavior [47].

The palatability of protein-enriched foods largely depends on the protein-carrier ‘fitness’. A precise selection of protein type and food carrier which could inhibit or even benefit from the sensory impacts caused by protein enrichment plays a vital role in developing appealing protein-enriched products. From a sensory point of view, in some cases, the mild flavor and taste of WPI made it more proper for protein-enrichment, as compared to WPH [48]. However, from a nutrition point of view, the nutritional value of WPH is relatively higher due to its higher digestibility and absorptivity than WPI [12,13], which makes it worthwhile to put efforts into broadening the use of protein hydrolysate through modifying its production and processing or identifying masking agents in order to improve its sensory quality [34,42,48]. 

However, the quality of protein ingredients used for enrichment, such as the digestion and absorption rate and amino acid compositions, might be partly affected by the production process of enriched foods. The potential quality changes may further influence the enriched foods’ contribution to muscle protein synthesis. Evaluations on the protein quality of enriched foods and clinical trials on the biological utilization of protein-enriched foods might be needed in future studies. 

In this study, screened trained panelists aged 23–49 years were used in the sensory descriptive analysis, and provided reliable and clear characterizations of prototypes. The use of older panelists of a similar age to the target consumers as part of the trained panel was considered, however, descriptive analysis with older panels may introduce more noise in characterizing products due to their highly heterogeneous sensitivity [38]. More investigations regarding the proper use of older panels are needed. With increasing age, adults are more receptive to functional foods because of their increased health considerations, especially in the prevention of chronic diseases [49]. In this context, appealing protein-enriched functional products hold a bright future in the market of older consumers. Older consumers had high prospective willingness towards consumption of protein-enriched rye bread and cream cheese in this study. However, besides ‘good taste’, there are a number of drivers and obstacles for consumption of protein-enriched foods. A better understanding of motivators for consumption of protein-enriched products among target consumers could help the promotion of protein intake.

In the present study, a lab-based consumer acceptance test was conducted to obtain a general perspective on how consumers accept the products. To evaluate consumer acceptance of protein-enriched foods or meals in real life, contextual aspects could be considered and included for exploration in future consumer studies to strengthen the predictive power of the results [50]. 

The most preferred enriched prototypes of the two food matrices had twice amount of protein as compared to non-enriched controls. Per slice, the WPH 7-TS bread contained 7.0 g protein, which was 4.0 g more than the non-enriched control bread (Table 1). Each serving of WPI 9-TS cream cheese contained 2.9 g protein, 1.8 g more than the control cheese (Table 3). Assuming that older adults could consume two to three slices of bread combined with two to three servings of cream cheese in one meal, the protein intake could increase by 11.6–17.4 g/meal due to protein-enrichment, achieving 19.8–29.7 g/meal in total, which is close to the dietary recommendation for older adults (25–30 g/meal) [8]. However, to evaluate the increase of protein intake through consumption of protein-enriched foods in real life, further studies are needed to investigate the effects of protein-enrichment on food intake and satiety in target older consumers [51]. 

## 5. Conclusions

The present study evaluated different kinds of protein-enrichments of rye bread and cream cheese for their sensory acceptability by independent senior citizens. Relationships between sensory properties of the protein fortification in these products were established. Sensory acceptability by senior consumers was different with respect to the sensory properties of appearance, flavor, and texture, indicating that diverse protein fortification strategies should be considered in product development and optimization to be able to satisfy and engage senior consumers in the consumption of such nutritious products.

## Figures and Tables

**Table 1:** Recipes of rye bread samples (per loaf).

Sample ^1^	Initial Dough ^2^ (g)	Protein Fortifier			Texture and Taste Modification			Total Weight before Baking (g)	Total Weight after Baking (g)	Total Protein Content (%)	Protein Content per Slice ^5^ (g)
		WPH (g)	WPI (g)	SPI (g)	Additional Water (g)	Wheat Gluten ^3^ (g)	Dried Sourdough ^4^ (g)				
Control	748	0	0	0	0	0	0	748	506.5	8.6	3.0
WPH 4	717.7	30.3	0	0	0	0	0	748	501.4	13.6	4.8
WPH 7	693.2	54.8	0	0	0	0	0	748	503.9	17.5	6.1
WPH 7-T	675.2	54.8	0	0	0	18	0	748	501.1	19.9	7.0
WPH 7-TS	660.2	54.8	0	0	0	18	15	748	500.9	20.0	7.0
WPI 4	717.7	0	30.3	0	0	0	0	748	498.4	13.7	4.8
WPI 7	693.2	0	54.8	0	0	0	0	748	503.9	17.5	6.1
WPI 7-T	675.2	0	54.8	0	0	18	0	748	504.5	19.8	6.9
WPI 7-TS	660.2	0	54.8	0	0	18	15	748	516.1	19.5	6.8
SPI 4	717.7	0	0	30.3	0	0	0	748	498.9	13.9	4.9
SPI 7	693.2	0	0	54.8	0	0	0	748	514.6	17.5	6.1
SPI 7-T	623.2	0	0	54.8	70	0	0	748	501.5	17.7	6.2
SPI 7-TS	612.7	0	0	54.8	70	0	10.5	748	515.2	17.3	6.1
WPI 4 + SPI 4	636.9	0	30.3	30.3	40	0	10.5	748	500.0	19.0	6.5
WPH 4 + SPI 4	636.9	30.3	0	30.3	40	0	10.5	748	502.7	18.8	6.6

^1^ Labels of the sample: WPH = whey protein hydrolysate; WPI = whey protein isolate; SPI = soy protein isolate; numbers in the labels indicate amount of added whey or soy protein (4 = 4%, and 7 = 7%); T = texture-modified samples; TS = texture and taste-modified samples. ^2^ Preparation of initial dough: First, 20 g yeast was dissolved in 800 mL water, which was then mixed with 1000 g rye bread mix (Amo, Glostrup, Denmark) and 50 g sunflower seeds, using hand mixer in medium speed for 10 min. Amo’s rye bread mix consists of rye flour, wheat flour, rye flakes, sunflower seeds, dried sourdough, salt, sugar, wheat starch, malt, and barley flour. It can also contain egg, milk, soy, and/or lupine. Amo’s rye bread mix contains 9.9% protein. The additional sunflower seeds contain 21.0% protein. ^3^ Wheat gluten (Naturkost Engros, Odense, Denmark) contains 71.0% protein. ^4^ Dried sourdough powder (KageButikken, Albertslund, Denmark) is made from rye flour and wheat flour and contains 10.0% protein. ^5^ Bread weight per slice: 35 g.

**Table 2:** Sensory attributes and corresponding definitions used in the descriptive analysis of rye breads.

Category	Attributes	Definitions
**Odor**	Yeasty	Odor associated with yeast fermentation in bread
	Malty	Odor associated with germinated cereal grains
	Burned	Odor associated with over-baked breads
***Crumb***		
**Appearance**	Brown	Degree of color brownness in the crumb, ranging from light brown to dark brown
	Compact	Appearance impression of the crumb density of the bread cross section
	Porosity	The extent of holes and cracks in the crumb of the bread cross section
**Mouthfeel**	Stickiness	The force needed to remove bread particles stuck to the palate completely
	Floury	Degree to which the crumb contains small grainy particles
	Astringent	The drying and puckering sensation evoked by strong black tea
**Texture**	Soft	Degree of yielding readily to pressure between palate and tongue
	Dry	Amount of saliva absorbed by sample crumbs during mastication
	Elasticity	The ability to resist force between palate and tongue and return to its original shape
	Crumbly	The force with which the sample crumbles
	Coarse	Degree to which particles abrade palate and tongue during mastication
**Flavor**	Buttermilk	Flavor impression of cultured buttermilk
	Beany	The off-flavor associated with soaked beans
	Grainy	Flavor impression of cereal derived rye grains, wheat grains etc.
**Taste**	Sweet	Basic taste evoked by sucrose
	Salty	Basic taste elicited by sodium chloride
	Bitter	Basic taste of quinine
	Sour	Basic taste evoked by citric acid
	Umami	Basic taste elicited by monosodium glutamate
	Balance	The perceived overall balance of five basic tastes
**After-taste**	Sour	Taste sensation evoked by citric acid
	Bitter	Taste sensation of quinine
***Crust***		
**Appearance**	Brown	Degree of color brownness in the crust, ranging from light brown to dark brown
**Texture**	Hardness	The force needed to bite through the bread crust completely between molars
**Taste**	Sour	Basic taste evoked by citric acid
**After-taste**	Bitter	Taste sensation of quinine

**Table 3:** Cream cheese recipes (per 100 g).

Sample ^1^	Cream Cheese ^2^ (g)	Protein Fortifier		Texture and Taste Modification ^3^	Total Weight (g)	Total Protein Content (%)	Protein Content per Serving ^4^ (g)
		WPH (g)	WPI (g)	Butter (g)			
Control	100	0	0	0	100	4.5	1.1
WPH 9	91	9	0	0	100	11.9	3.0
WPI 9-TS	81	0	9	10	100	11.6	2.9
WPH 9	91	9	0	0	100	11.9	3.0
WPH 9-TS	81	0	9	10	100	11.6	2.9

^1^ Labels of the sample: numbers in the labels indicate the amount of added protein (9 = 9%); TS = texture and taste-modified samples. ^2^ Arla Buko ^®^ Natural Cream Cheese (Arla Foods, Viby J, Denmark) contains 4.5% protein, 25% fat, and 0.5% salt. ^3^ Butter was weighed and softened in room temperature for 0.5 h before being mixed with cream cheese, using a hand mixer at slow speed for 1 min. The butter contains 1.0% salt and 0.9% protein. ^4^ Weight per serving: 25 g.

**Table 4:** Sensory attributes and corresponding definitions used in the descriptive analysis of cream cheese.

Category	Attributes	Definitions
**Odor**	Butter	Odor associated with softened butter
**Appearance**	Yellow	Degree of color yellowness in the surface of sample
	Glossy	Degree to which the surface of cream cheese is shiny
**Texture**	Smooth	Absence of any particles or lumps in the sample
	Firmness	Extent of resistance against the palate and tongue during mastication
	Meltdown rate	The amount of “work” required to break down the bolus
	Viscosity	Stickiness between tongue and upper palate
**Mouthfeel**	Astringent	The drying and puckering sensation evoked by strong black tea
	Coating	Extent to which the cheese coats the palate and tongue during mastication
**Flavor**	Creamy	Flavor associated with whipped cream
	Buttermilk	Flavor impression of cultured buttermilk
	Fatty	Flavor associated with butter
	Egg yolk	Flavor associated with cooked egg yolk
	Rancid	Flavor associated with oxidized, rancid cooking oil
	Fresh cheesy	Flavor associated with fresh, mild cheese without mold flavor, e.g., fresh mozzarella or ricotta
**Basic taste**	Salty	Basic taste elicited by sodium chloride
	Bitter	Basic taste of quinine
	Sour	Basic taste evoked by citric acid
	Sweet	Basic taste evoked by sucrose
	Umami	Basic taste elicited by monosodium glutamate
**After-taste**	Bitter	Taste sensation of quinine

**Table 5:** Mean ratings of consumers’ liking for rye bread samples. The size of each cluster is indicated (%).

Sample	Cluster 1 (24%)	Cluster 2 (50%)	Cluster 3 (26%)	Mean (100%)
Control	6.7abA	6.8aA	5.9bA	6.5A
SPI 7	5.8aB	5.9aB	4.4bB	5.5C
WPI 7	5.8aB	4.7bC	6.5aA	5.5C
WPH 7	5.5BC	5.6B	5.7A	5.6BC
WPH 7-TS	4.8bC	6.3aAB	6.6aA	6.0AB
WPH 4+ SPI 4	6.3A	5.7B	6.1A	5.9BC

Different lowercase letters within the same row indicate significant post hoc Fisher’s least significant difference (LSD) differences at *p* < 0.05; different capital letters within the same column indicate significant LSD differences at *p* < 0.05.

**Table 6:** Mean ratings of consumers’ liking towards cream cheese. Size of each cluster was indicated (%).

Sample	Cluster 1 (68%)	Cluster 2 (24%)	Cluster 3 (8%)	Mean (100%)
Control	6.2abB	7.1aA	4.8bAB	6.3AB
WPI 9	6.4aB	5.8abBC	4.5bB	6.1BC
WPI 9-TS	7.2aA	6.8aAB	5.0bAB	6.9A
WPH 9	5.1bC	6.9aA	5.5abAB	5.6C
WPH 9-TS	5.4C	5.5C	6.7A	5.5C

Different lowercase letters within the same row indicate significant LSD differences at *p* < 0.05; different capital letters within the same column indicate significant LSD differences at *p* < 0.05.
